# Associations Between Sleep and Mental Health Among Latina Adolescent Mothers: The Role of Social Support

**DOI:** 10.3389/fpsyg.2021.647544

**Published:** 2021-05-21

**Authors:** Shun Ting Yung, Alexandra Main, Eric A. Walle, Rose M. Scott, Yaoyu Chen

**Affiliations:** Department of Psychological Sciences, University of California, Merced, Merced, CA, United States

**Keywords:** Latinx, adolescent mothers, sleep, social support, mental health

## Abstract

Adolescent mothers experience poorer sleep than adult mothers, and Latina adolescent mothers are at greater risk of postpartum depression compared with other racial/ethnic groups. However, social support may be protective against the negative effects of poor sleep in this population. The current study examined (1) associations between the quality and quantity of Latina adolescent mothers’ sleep and mental health (depressive symptoms and anxiety), and (2) whether social support buffered the effects of poor sleep on mental health. A sample of Latina adolescent mothers (*N* = 84) from an agricultural region in the United States reported on their sleep duration/quality, social support from family, friends, and significant others, and their depressive and anxiety symptoms. Results showed that adolescent mothers reported poorer sleep than pediatric recommendations, and poorer sleep quality was associated with greater depressive and anxiety symptoms. Interestingly, when adolescent mothers reported better sleep, they had fewer depressive symptoms in the context of high support from friends compared with low support from friends. Sleep is important for mental health in Latina adolescent mothers, and better sleep combined with strong social support has positive associations with mental health in this population. Findings hold implications for improving mental health in adolescent mothers.

Adolescent mothers face unique challenges in their transition to motherhood, including higher levels of depression compared with adult mothers (Lanzi et al., 2009). Adolescent mothers are more likely than adult mothers to live in poverty, particularly in agricultural regions of the United States such as the San Joaquin Valley in California where the adolescent birthrate is nearly twice the overall rate statewide (California Department of Public Health, 2017). Though adolescent pregnancy rates are generally declining, adolescents of color experience greater health disparities and are at higher risk for unplanned pregnancies (Office of Disease Prevention and Health Promotion, n.d.), particularly Latina adolescents (Jahromi et al., 2012). Thus, it is crucial to understand factors that (a) are associated with Latina adolescent mothers’ mental health, and (b) may buffer Latina adolescent mothers from poor mental health outcomes.

## Challenges of Adolescent Motherhood

Adolescent mothers are more likely to experience psychological and behavioral adjustment problems than adult mothers (Chang and Fine, 2007). The need to integrate their maternal role with their individual identity development can create role conflict, confusion, and emotional distress (Birkeland et al., 2005). Over half of adolescent mothers experienced moderate to severe depressive symptoms during the first 4 years postpartum (Schmidt et al., 2006). Compared to European American mothers, Latina adolescent mothers are more vulnerable to depression (Howell et al., 2005). Sociocultural stressors, including discrimination and acculturative stress experienced during the perinatal period, may contribute to poor mental health in this population (e.g., Luis Sanchez et al., 2020). Furthermore, Latina mothers living in rural communities have a 40% greater chance of developing postpartum depression compared to White mothers (Ceballos et al., 2016). However, risk and protective factors in the context of Latina adolescent mothers’ mental health remain understudied.

## Postpartum Sleep and Mental Health

Sleep is intricately linked with physical, cognitive, and psychological health and wellbeing (e.g., Sadeghi Bahmani et al., 2018; Zhang et al., 2018). New mothers are often vulnerable to the negative effects of sleep disruptions during the postpartum period (Richter et al., 2019). Insufficient sleep is related to greater psychological distress and vulnerability to mood disorders, including anxiety, and depression (e.g., Okun et al., 2018). Researchers have found that depressive symptoms at 1-month postpartum predict worse sleep quality at 6 months, which predicts greater depressive symptoms at 6 and 12 months postpartum (Stremler et al., 2019). Disrupted sleep among postpartum mothers can develop into a “vicious cycle” of poor sleep quality and depression.

The National Sleep Foundation in the United States recommends 8–10 h of sleep per night for adolescents ages 14–17 (Hirshkowitz et al., 2015). Adolescent mothers may be at particular risk for poor sleep because adolescents with insomnia have greater difficulty with affective regulation (Brand et al., 2016), which has implications for poor mental health, including depression and anxiety (Zhang et al., 2017). Conversely, longer sleep duration is associated with better subjective psychological well-being among adolescents (Kalak et al., 2014). Adolescents also undergo critical biological changes in sleep patterns, including sleep phase delay, a natural process in which adolescents have a tendency to go to sleep and wake later (Shochat et al., 2014). In addition to biological changes, adolescence coincides with changing environmental factors (e.g., earlier school start times, growing academic workload, less parental influence on bedtime, increased technology usage), which may contribute to decreased sleep, and in turn increase anxiety and depression (Shochat et al., 2014). Taken together, adolescent mothers may experience more mental health problems caused by sleep disruptions than adult mothers due to these normative changes. Despite these risks, the impact of sleep on adolescent mothers’ mental health has not been examined to our knowledge.

## The Role of Social Support

Social support from close others is an important protective factor against postpartum depression (Hahn-Holbrook et al., 2018), and may be particularly important for adolescent mothers’ mental health (Oberlander et al., 2007). Previous studies have shown that social support predicts less depression among adolescent mothers (see Reid and Meadows-Oliver, 2007). Social support may be especially important for Latina adolescent mothers, as family support is central to Latinx culture (e.g., Bravo et al., 2016). For example, Latina mothers are more likely to seek support and share childcare responsibilities with family members compared to other ethnic groups (Cauce et al., 2002). Furthermore, high-quality family interactions are related to healthy sleep among Latinx adolescents (Sasser et al., 2020). Indeed, Latina adolescent mothers who live with family members experience lower parenting stress compared to their African American peers (Huang et al., 2019).

While family support may protect against negative outcomes for adolescent mothers, peers may also be an important source of support. For example, a case study showed that an adolescent mother reported greater conflict with parents and believed friends were more helpful than parents (Stiles, 2010). Moreover, the involvement of the child’s father or the mother’s significant other is an important source of support for adolescent mothers (Smith et al., 2013). Latina adolescent mothers report less postpartum depression when fathers are more involved with childcare (Fagan and Lee, 2010). Taken together, support from parents, friends, and significant others may all be important protective factors for adolescent mothers. However, to our knowledge, prior research has not investigated how social support may buffer the effects of poor sleep on mental health postpartum.

## The Present Study

The present study examined sleep, mental health, and social support in a sample of Latina adolescent mothers from an agricultural, predominantly rural region of the United States. The aims of the present study were to examine: (1) the quality and quantity of Latina adolescent mothers’ sleep, (2) associations between sleep and mental health (depressive symptoms and anxiety), and (3) whether social support from family, friends, and significant others buffered the effects of sleep duration/quality on mental health. We hypothesized that adolescent mothers would have lower sleep quality and quantity compared with pediatric recommendations, and that lower quality/quantity of sleep would be associated with higher depressive symptoms and anxiety. We also hypothesized that social support from family, friends, and significant others would buffer the effects of sleep duration/quality on mental health among adolescent mothers. Given the unique demographics of the sample, we also explored associations between demographic characteristics (primary caretaker education and generation status) and sleep, mental health, and social support.

## Materials and Methods

### Participants

Participants were 84 adolescent mothers, *M*_age_ (*SD*) = 17.81 (0.94) years, who self-identified as Latina. Adolescents were eligible to participate if they were between 13 and 19 years of age, were a biological parent (*N* = 73, 45% of children = female) or pregnant (*N* = 11) at the time of participation and could read or speak English or Spanish. Participants were recruited from high schools and afterschool programs in California’s San Joaquin Valley. The majority of participants were born in the United States (81%), followed by Mexico (18%), or another country (1%). Participants reported that their mother was born in the United States (47%), Mexico (52%), or another country (1%), and that their father was born in the United States (38%), Mexico (59%), or another country (3%). Over half of the adolescent mothers were single (63%) at the time of participation, 33% were living with their spouse or romantic partner, and 1% were married. The median education level of the adolescent’s primary caretaker was a high school diploma or equivalent (see Table 1 for more descriptive information about the sample).

**TABLE 1:** Descriptive statistics of demographic, sleep, social support, and mental health variables.

**Variable**	**Min**	**Max**	**Mean**	***SD***
Adolescent age	14.88	19.88	17.81	0.94
Child age (months)	0.79	48.20	13.42	9.87
Primary caretaker education^a^	1.00	4.00	1.53	0.79
Generation status^b^	0.00	2.00	1.10	0.74
Sleep quantity (hours)	2.00	10.00	6.55	1.80
Sleep quality	1.00	5.00	3.05	1.07
Depressive symptoms	0.00	26.00	11.57	6.09
Anxiety symptoms	20.00	58.00	35.55	8.65
Family support	1.00	7.00	4.87	1.69
Friend support	1.00	7.00	4.13	2.18
Significant other support	1.00	7.00	5.24	1.70

*Min, minimum; Max, maximum, SD, standard deviation. ^*a*^Primary caretaker education is coded as: 1 = some high school or less, 2 = high school graduate or equivalent, 3 = some college, 4 = associates/vocational degree, 5 = bachelors degree, 6 = masters degree, 7 = MD/PhD/JD. ^*b*^Generation status is coded as: 0 = adolescent born outside the United States, 1 = at least one parent born outside the United States, and 2 = both parents born in the United States.*

### Procedure

The study was approved by the Institutional Review Board at the academic institution where the research was conducted. Parents provided informed consent for adolescents less than 18 years of age and adolescents provided assent (if under 18) or consent (if 18 or older). Participants completed either an online survey or a paper survey that was administered by trained researchers in a classroom setting. Questionnaires not previously used in research with Spanish-speaking samples were forward and back-translated by bilingual research assistants.

### Measures

#### Sleep Duration/Quality

Questions derived from the Pittsburgh Sleep Quality Index (PSQI; Buysse et al., 1989) were used to access adolescents’ sleep quality and duration. This measure has good test-re-test reliability and diagnostic sensitivity with adults and adolescents (Buysse et al., 1989; Raniti et al., 2018). Participants were asked to report on the quantity and quality of sleep in the past month by responding to the following questions: (1) “On average, how many hours of sleep do you get per night?” (1 = *less than 4 h* to 5 = *more than 9 h*), and (2) “How would you describe the quality of your sleep on an average night?” (1 = *very poor* to 5 = *very good*). The items were highly correlated (*r* = 0.62, *p* < 0.001); thus, a composite was created by standardizing the items and computing the mean. This variable was used in all analyses of sleep.

#### Depressive Symptoms

The 11-item version of the Center for Epidemiologic Studies Depression Scale (CES-D; Radloff, 1977) assessed adolescent depressive symptoms in the past week. The CES-D is a commonly used self-report measure of depressive symptoms that has been shown to have high internal consistency and validity in adolescent samples (e.g., Radloff, 1977; Stockings et al., 2015), including Latinx adolescents (Suarez-Colorado et al., 2020). Each item was rated on a scale of 0 (*rarely or none of the time*) to 3 (*most or all the time*). Sample items include, “I did not feel like eating; my appetite was poor” and “I felt everything I did was an effort.” Scores were summed to create an overall depressive symptoms score, with a higher total score indicating more depressive symptoms. This scale demonstrated adequate internal consistency (α = 0.75).

#### Anxiety

The 20-item State-Trait Anxiety Inventory for Children (STAI-C; Spielberger et al., 1973) assessed adolescent anxiety in the past month. The STAI-C is a widely used tool to evaluate the presence and severity of current anxiety symptoms. The STAI-C has good internal consistency and high convergent and discriminative validity (Bakker et al., 1989; Beesdo et al., 2009) and is a valid measurement of anxiety in the postpartum period (Dennis et al., 2013). Each item was rated on a scale of 1 (*hardly ever*) to 3 (*often*). Sample items include, “It is difficult for me to face my problems” and “I am secretly afraid.” Scores were summed to create an overall anxiety score, with a higher total score indicating more anxiety. The internal consistency coefficient was α = 0.90.

#### Social Support

The 12-item Multidimensional Scale of Perceived Social Support (Zimet et al., 1988) assessed social support from family, friends, and significant others. Each item was rated on a scale of 1 (*very strongly disagree*) to 7 (*very strongly agree*). Sample items include, “There is a special person who is around when I am in need” and “There is a special person in my life who cares about my feelings.” This measure has been shown to be a suitable measure of perceived social support in Latinx adolescents (Trejos-Herrera et al., 2018). Scores were summed separately for the three sources of social support in the current study (α = 0.89, 0.95, and 0.92 for family, friends, and significant other, respectively).

## Results

Descriptive statistics for demographic and study variables are presented in Table 1. Variables were screened for normality; all fell within the normal range of 2 for skewness and 7 for kurtosis. Participants reported an average of 6.5 h of sleep per night (*SD* = 1.80 h), with nearly 82% of the sample reporting fewer than 8 h of sleep per night. This is lower than the recommended 8–10 h and average 9 h of sleep for adolescents (Hirshkowitz et al., 2015). Moreover, nearly one third of the sample reported very poor or somewhat poor quality of sleep.

Zero-order correlations were conducted to examine relations among the demographic and study variables (see Table 2). There were no significant associations between demographic variables and sleep, mental health, or social support; thus, these variables were not included in subsequent analyses. Adolescents who reported poorer sleep reported higher depressive and anxiety symptoms. Additionally, adolescents who reported receiving more family support indicated receiving more support from friends and their significant others. There were no significant associations between social support and sleep or mental health.

**TABLE 2:** Correlations between demographic variables, sleep, social support, and mental health variables.

**Variable**	**1**	**2**	**3**	**4**	**5**	**6**	**7**	**8**	**9**	**10**
1. Adolescent age	–									
2. Child age (months)	0.32**	–								
3. Child gender (1 = female, 2 = male)	–0.03	0.06	–							
4. Primary caretaker education	0.08	–0.19	–0.05	–						
5. Generation status	0.06	–0.13	0.01	0.18	–					
6. Adolescent sleep	–0.13	–0.09	0.04	0.00	–0.13	–				
7. Depressive symptoms	0.08	0.09	–0.02	–0.11	–0.15	−0.35**	–			
8. Anxiety symptoms	0.02	–0.04	–0.07	0.23	0.13	−0.46**	0.57**	–		
9. Family support	0.06	–0.14	–0.08	0.05	–0.04	0.21	–0.08	–0.05	–	
10. Friend support	0.04	–0.10	0.01	–0.07	0.04	0.17	–0.12	–0.04	0.49**	–
11. Significant other support	0.07	–0.12	–0.10	0.09	–0.27	0.03	–0.16	–0.03	0.61**	0.54**

**p < 0.01, *p < 0.05.

To test whether social support buffered the effects of poor sleep on depressive and anxiety symptoms, 6 hierarchical multiple regression analyses were conducted with social support from each source (family, friends, and significant other) included in the models along with the interaction with sleep duration/quality. The first step of each model included the main effects of sleep and social support. The second step included the main effects and the interaction term of sleep and social support. Interaction terms were created by centering both variables around their mean and multiplying them together.

**FIGURE 1:**
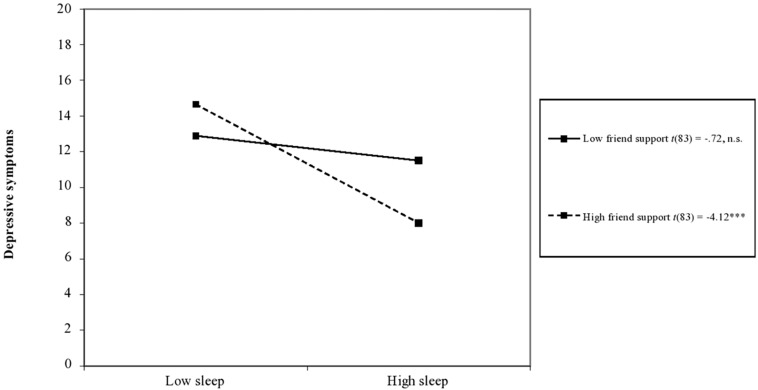
Interaction between sleep duration/quality and social support from friends predicting adolescent parents’ depressive symptoms. *Notes: t*-values are unstandardized simple slopes. *n.s.*, not significant, ***p < 0.001.

Results revealed a main effect of sleep for all models, with better sleep associated with fewer depressive and anxiety symptoms (see Table 3). Results also revealed a significant interaction between sleep and social support from friends predicting depressive symptoms (*B* = –0.67, *p* = 0.03). Plotting of the interaction using simple slopes analysis showed that adolescents reported fewer depressive symptoms when sleep duration/quality was high compared with poorer sleep, but only if support from friends was also high (see Figure 1). No other significant interactions between social support and sleep predicting mental health were present.

**TABLE 3:** Standardized hierarchical multiple regressions predicting depressive and anxiety symptoms from social support and sleep duration/quality.

	**Depressive symptoms**		**Anxiety symptoms**	
**Variable**	**β**	**Δ*R*^2^**	β	**Δ*R*^2^**
Step 1		0.10		0.19
Family support	–0.02		0.05	
Sleep	−0.34*		−0.47*	
Step 2		0.09		0.19
Family support × Sleep	–0.02		0.08	
Step 1		0.10		0.21
Friend support	–0.07		0.04	
Sleep	−0.33*		−0.46*	
Step 2		0.14		0.21
Friend support X Sleep	−0.22*		–0.07	
Step 1		0.12		0.19
Significant other support	–0.15		–0.02	
Sleep duration/quality	−0.34*		−0.45*	
Step 2		0.11		0.18
Significant other support × Sleep	0.05		–0.02	

*p < 0.05.

## Discussion

The current study fills an important gap in the literature by examining relations between sleep duration and quality, social support (family, friends, and significant other) and mental health (depressive and anxiety symptoms) in Latina adolescent mothers in a predominantly rural, agricultural region of the United States. Findings demonstrated that fewer hours of sleep and lower quality of sleep were linked with higher depressive symptoms and anxiety, and that social support from friends buffered the association between poor sleep and depressive symptoms. These findings are discussed in detail below.

### Sleep, Mental Health, and Social Support

In line with previous studies, adolescent mothers in the current sample were at risk for inadequate sleep. The average number of hours of sleep in the past month for the sample in the current study is lower than pediatric recommendations (8–10 h of sleep per night; Hirshkowitz et al., 2015), and considerably lower than the average number of hours of sleep reported in a large study of Canadian adolescents, which found that approximately one-third of adolescents reported insufficient sleep (Chaput and Janssen, 2016). It is common for new mothers to report insomnia, sleep deficiency or sleep disturbances (e.g., Reichner, 2015). This may be particularly true for adolescent mothers because adolescence is a critical developmental period when sleep patterns undergo significant changes (Shochat et al., 2014).

Consistent with hypotheses, adolescent mothers who reported worse sleep (fewer hours and lower quality) reported higher depressive symptoms and anxiety. This is consistent with previous studies with adult mothers (e.g., Figueiredo et al., 2007) and findings that inadequate sleep is associated with depression and anxiety in adolescence (e.g., Shochat et al., 2014). The current study extends these findings to a sample of Latina adolescent mothers, who are at particular risk of mental health problems in the postpartum period (Bámaca-Colbert et al., 2011).

We found partial support for our second hypothesis that social support would buffer the effects of poor sleep on adolescent mothers’ mental health, but only for social support from peers. Specifically, adolescents reported fewer depressive symptoms in the context of both better sleep and higher social support from friends. Growing reliance on peers rather than parents for social support is an important transition in adolescence (Brown and Larson, 2009), particularly when conflict with parents is high (Stiles, 2010). However, social support from family and significant others did not buffer the effects of poor sleep on mental health. These findings underscore the importance of considering multiple biological and environmental factors that undergo change during adolescence.

Somewhat surprisingly, primary caretaker education and generation status (i.e., whether adolescent mothers were born in the United States) were not associated with participants’ quality of sleep, mental health or social support. Low socioeconomic status has been identified as a mental health risk factor for first-time mothers (Goyal et al., 2010), and prior research has shown that U.S.-born Latinx youth and those with U.S.-born parents are more likely than their non-U.S.-born counterparts to engage in risky behaviors, including tobacco, drug, and alcohol use and have earlier age of sexual initiation (e.g., Kaplan et al., 2002). Future research could explore other cultural factors, such as cultural values (e.g., *familismo*) and acculturative processes, to test whether maintenance of traditional cultural values or acculturation are linked with social support and may buffer Latina adolescent mothers from poor mental health outcomes.

### Limitations, Future Directions for Research, and Clinical Implications

The current study is an important first step in understanding the role of sleep in Latina adolescent mothers’ mental health. However, some limitations warrant mentioning. First, the current study relied on self-report and assessed study variables retrospectively, which may be subject to bias. Future research could use more objective measures of sleep, including sleep tracking devices and observed measures of relationship quality between adolescent mothers and social support figures. Second, the cross-sectional design precludes examining developmental changes in associations between adolescent mothers’ sleep quality and mental health. Finally, though the current study sheds light on associations between sleep, social support, and mental health within an understudied population (i.e., Latina adolescent mothers), comparisons to other groups (e.g., other ethnicities, urban samples) would help identify potentially unique protective factors within this population.

Our findings have important implications for future research directions. It is important to stress the multifaceted impact of affective disorders associated with reduced sleep quality. For example, postpartum depression has been found to have negative consequences for the quality of mother-child interactions (Slomian et al., 2019). These consequences include poor physical and psychological health, increased risk behaviors, and worsened interpersonal relationships for mothers. Moreover, maternal postpartum depression also has negative consequences for a host of infant outcomes due to reduced caregiving sensitivity, including physical, behavioral, cognitive, linguistic, and socioemotional development (e.g., Grace et al., 2003; Lanzi et al., 2009; Slomian et al., 2019). The broad-reaching impact of adolescent mothers’ mental health on their children and social networks is an important topic for future investigation.

Finally, this study holds implications for understanding sleep and mental health in Latina adolescent mothers. Healthcare practitioners should be aware of the importance of adequate sleep for adolescent mothers and assess postpartum sleep duration and quality. Our findings also indicate the importance of peer support that adolescent mothers receive. Interventions focused on reducing psychological distress in adolescent mothers should consider ways to strengthen their support from friends and peer network to promote positive mental health in this at-risk population.

## Data Availability Statement

The raw data supporting the conclusions of this article will be made available by the authors, without undue reservation.

## Ethics Statement

The studies involving human participants were reviewed and approved by The UC Merced Institutional Review Board, holding Department of Health and Human Services Federalwide Assurance #00005105, has reviewed and approved this study. It was approved by Ramesh Balasubramaniam, the Chair of the IRB. Written informed consent to participate in this study was provided by the participants’ legal guardian/next of kin. Participants who were 18 years old or above provided informed consent themselves.

## Author Contributions

SY, AM, EW, and RS performed the material preparation and data collection and analysis. SY wrote the first draft of the manuscript. YC contributed to the literature review. All authors commented on previous versions of the manuscript, read and approved the final manuscript.

## Conflict of Interest

The authors declare that the research was conducted in the absence of any commercial or financial relationships that could be construed as a potential conflict of interest.
